# Risk Factors for Cardiovascular Diseases among Diabetic Patients In Southwest Ethiopia

**DOI:** 10.4314/ejhs.v20i2.69438

**Published:** 2010-07

**Authors:** Solomon Tamiru, Fessahaye Alemseged

**Affiliations:** 1 Department of Internal Medicine, Jimma University; 2 Department of Epidemiology, Jimma University

**Keywords:** Diabetes Mellitus, Hypertension, Obesity, Dyslipidemia, Ethiopia

## Abstract

**Background:**

Studies on cardiovascular risk factors among diabetic persons in Ethiopia are lacking. The objective of this study was to determine the prevalence of the cardiovascular risk factors (hypertension, obesity, physical inactivity, dyslipidemia and smoking) among diabetic patients at the diabetic clinic of Jimma University Specialized Hospital.

**Methods:**

A cross-sectional study was conducted from October to December 2007. Three hundred one individuals were randomly selected from 950 patients on follow-up. Data were collected using a structured format and appropriate equipments and reagents. Laboratory data were recorded in a separate checklist. The data were entered into SPSS for Windows version 12. Multivariate regression analysis was carried out to identify predictors of hypertension, obesity and dyslipidemia.

**Results:**

Two hundred and fifty six (85.1%) of the sample participated in the study. The prevalence of hypertension, obesity, dyslipidemia, physical inactivity and current smoking was 46.5%, 23.4%, 63.5%, 55.1% and 5.5% respectively. Age ≥ 45 years, type 2 diabetes and obesity were predictors of hypertension. Females were less likely to be hypertensive (OR =2.26, 3.37, 3.79 and 0.48 respectively). Type 2 diabetics and females were more while rural diabetics were less likely to be obese. (OR =6.08, 4.17 and 0.37 respectively). Female gender, hypertension and fasting blood glucose ≥ 180mg/dl were predictors of dyslipidemia. Alcohol users were less likely to be dyslipidemic. (OR =4.25, 3.5, 3.56 and 0.39, respectively)

**Conclusion:**

Hypertension, obesity, dyslipidemia and physical inactivity were common while smoking was uncommon among diabetic patients in Jimma University Specialized Hospital. Type 2 DM was a predictor of hypertension and obesity. Diabetic women were more likely to be obese and dyslipidemic. We recommend screening and management of these risk factors.

## Introduction

The global burden of diabetes mellitus is rising dramatically. There are 230 million diabetic patients worldwide; two thirds of whom are in developing countries. By the year 2030, more than 80% of the 366 million diabetic patients will be in developing countries (1). In Ethiopia, national data on prevalence and incidence of diabetes are lacking. However, patient attendance rates and medical admissions in major hospitals are rising (2, 3). A population based study in northwestern Ethiopia (Gondar) showed an overall prevalence of diabetes and impaired glucose tolerance of 0.5%; a surprising low prevalence could be because most of the subjects were young (86%). Furthermore the prevalence of diabetes among older subjects (age > 40 years) was higher (2.4%) (4). Moreover, Cohen *et al* reported a high prevalence of diabetes (8.9%) among young (age < 30 years) Ethiopian Jews who have been to Israel for less than 4 years (5).

Cardiovascular diseases, i.e. Coronary heart diseases, stroke, and peripheral vascular diseases account for the majority of deaths in diabetic patients (6). Diabetes mellitus, hypertension, cigarette smoking, dyslipidemia, obesity and physical inactivity are established risk factors for cardiovascular diseases (CVD). These risk factors are known as traditional or conventional cardiovascular risk factors (6, 7). The conventional cardiovascular risk factors have greater impact on diabetic patients than non diabetics (6, 8). The multiple risk factors intervention trial (MRFIT) showed the presence of any one, two or all three risk factors (hypertension, cigarette smoking, dyslipidemia) increased the risk of CVD death more in diabetics than non-diabetic men (RR of 4.8, 4.0 and 2.6, respectively) (8).

Hypertension is common in diabetic patients especially type 2 diabetics (6, 9, 10, 11). There are variations in the prevalence of hypertension depending on the population and definition of hypertension (9, 10, 11). Institution based studies in Addis Ababa and Jimma reported that about a third of diabetics are hypertensive (12, 13). Dyslipidemias (abnormal lipoprotein levels) are common in type 2 (6, 9, 10, 11) but not in type 1 diabetics if the latter are under good glycemic control (6, 14). Obesity is also more common among type 2 diabetics (9) and is associated with dyslipidemia and hypertension (6, 15). In the west, up to 25% of diabetic patients smoke cigarettes (9, 10).

In Ethiopia, studies on the cardiovascular risk factors and complications of diabetes are lacking. A retrospective study in Tikur Anbesa Hospital showed that CVDs were responsible for 16% of deaths among diabetic admissions 2^nd^ to acute complications and infections that caused 18% of deaths (3).

In view of the growing burden of diabetes and its cardiovascular complications in the developing world, it is crucial to determine the physical and metabolic characteristics of diabetic persons in this part of the world. This study was conducted to assess the prevalence of cardiovascular risk factors among diabetic patients on follow-up at the diabetic clinic of Jimma university specialized hospital.

## Methods and Materials

This cross-sectional study was conducted at the diabetic follow-up clinic of Jimma University Specialized Hospital (JUSH) located in Jimma City, 357 km southwest of Addis Ababa. The hospital is a referral centre for the South-Western part of Ethiopia. The study was carried out from October to December 2007.

The study population was all adult (≥18 years) diabetic patients who were on follow-up at the diabetic clinic of JUSH registered till June 31, 2007. Pregnancy and acute illness were the exclusion criteria.

The sample size was determined using EPI-Info statistical software for estimating single population proportion. A total of 950 diabetic patients constituted the source population. The assumptions considered during calculation of the sample size were: 50% prevalence of CVD risk factors, 95% confidence level and margin of error of 5%. The final sample size with 10% non-response was 301. The patient registration book contains all diabetic patients ever enrolled for follow up at the clinic. The serial numbers of the patients who were on follow up, eligible, and not involved in the pretest were taken and the sample drawn using the lottery method.

Data were collected using a pretested structured format and a separate checklist for laboratory results. The format and checklist were linked by unique identification code (ID).

The data collection format had four parts. The first section was concerning socio-demographic characteristics. The second section was about habits including cigarette smoking, alcohol consumption, coffee drinking, exercise and diet. The third part was for clinical data including age at diagnosis, duration and treatment (current and initial) of diabetes; history of hypertension and its treatment. In the final part weight, height, BMI, waist circumference, and blood pressure were recorded. Weight was measured in kilograms (kg) using the WHO weighing scale (Health-O-Meter, USA) at a precision of 0.1kg with the study subjects minimally dressed. Height was measured in centimeter (cm) in erect position at a precision of 0.1cm with shoes removed. Waist circumference was measured in cm at the midpoint of the line between the lowest border of the thoracic cage and anterior superior iliac spine. Blood pressure was measured using a mercury sphygmomanometer with a cuff deflation rate of 2mmHg. Measurements in each arm after 10 min in sitting position were averaged to be recorded.

For laboratory measurements, blood was taken at the clinic after an overnight fasting of 10–14 hours. The patients attending the clinic were advised to come after an overnight fasting for the determination of FBG and lipid profiles. Urine samples were also obtained. FBG, serum total cholesterol, HDL-C and triglyceride were determined using the hemanalyzer machine (Human, USA) and appropriate reagents. LDL-C was calculated using the Freidwald formula (6, 14). Urine dipstick was done for presence of albuminuria.

The interview, chart review and physical measurement were carried out by trained medical interns. Laboratory samples were collected by an experienced nurse working at the diabetic clinic. Laboratory analyses were carried out by a laboratory technician. A first year medical resident supervised the data collection process at the clinic. The laboratory data collection and analysis was supervised by a senior laboratory technologist.

To ensure quality, pre-test was conducted on 30 patients, training was given to data collectors and supervisors on the data collection process and the collected data were checked for completeness and consistency on the day of collection.

The collected data were entered in to computer and analyzed using SPSS for windows version 12.1. The prevalence of hypertension, obesity, dyslipidemia, smoking and physical inactivity was determined. We employed multivariate regression to identify predictors of hypertension, obesity, and dyslipidemia.

During data analysis some variables were categorized into meaningful categories. Type 1 DM was defined as age at onset of diabetes < 30 years or insulin treatment from diagnosis. Type 2 DM was defined as age at onset ≥ 30 years and initial treatment with oral agents (6). Systemic hypertension, obesity, dyslipidemia and smoking were the conventional risk factors for CVD (6, 7). Hypertension was defined as systolic blood pressure (SBP) ≥ 140mmHg or diastolic blood pressure ≥ 90 mmHg or being on treatment for a physician diagnosed hypertension. Obesity was defined as BMI ≥ 30kg/m^2^ or waist circumference ≥ 102 cm for men and ≥ 88 cm for women. A patient was considered to have Dyslipidemia in the presence of at least 1 of the following: high plasma total cholesterol (≥200mg/dl), high LDL-C (≥130mg/dl), low HDL-C (<40mg/dl in men or < 50mg/dl in women), high triglyceride level (≥150mg/dl) (16, 17). A person who smokes any quantity of cigarette in the last 12 months was labeled as current smoker (7). A person who reports regular aerobic exercise (walking, jogging) of at least 30 min for every 5 days or its equivalent; or whose occupation requires physical exertion daily was considered to be physically active. (17). Macroalbuminuria was defined as detection of albumin in the spot urine specimen (6).

The study was approved by the ethical clearance committee of Jimma University. Each participant was informed in detail and his/her consent was obtained before data collection. Patients who were identified to have hypertension were informed and managed accordingly.

## Results

Out of the 301 randomly selected study subjects, 256 (85.1%) participated in the study.

The mean age of the study participants was 45.3 ± 14.6 years and 125 (48.9%) were younger than 45 years. A hundred and fifty nine (62.1%) were men and 153 (59.8%) of the respondents were urban residents. Eighty nine (34.8%) were illiterate while 102 (39.8%) had primary education. Sixty eight (26.6%) were farmers (Table 1).

**Table 1:** Baseline characteristics of diabetic patients on follow up at JUSH, Oct – Dec, 2007.

Variables		Number (n=256)	Percent
Socio-demographic characteristics	Age < 45 years	125	48.9
	> 45 years	131	51.1
	Gender Male	159	62.1
	Female	97	37.9
	Address Urban	153	59.8
	Rural	103	40.2
	Education	89	34.8
	Illiterate	102	39.8
	Grade 1–8	44	17.2
	Grade 9–12	21	8.2
	Tertiary		
	Occupation	68	26.6
	Farmer	73	28.5
	House wife	34	13.3
	Employee	22	8.6
	Merchant	20	7.8
	Laborer	14	5.5
	Pensioner	25	9.8
	Others		
Other baseline variables	Alcohol use	193	75.4
	No	63	24.6
	Yes		
	Chat chewing	195	76.8
	No	61	23.2
	Yes		
	Diabetes Type 1	85	33.2
	Type 2	171	66.8
	DM Duration < 5 (in years) 5-	129	50.4
	9.9	82	32.0
	> 10	45	17.6
	Current treatment		
	Oral	125	48.8
	Insulin	131	51.2
	FBG (mg/dl) < 126	66	25.8
	126–179.9	79	30.4
	180–249.9	69	27.0
	≥ 250	42	16.4
	
	Albuminuria No	157	61.3
	Yes	99	38.7

Type 2 diabetes accounted for 171 (66.8%) and the rest were type 1 diabetics. The mean duration of diabetes was 5.83 ± 5.42 years. In 129 (50.4%) of the study subjects, duration of diabetes was less than 5 years while only 45 had diabetes for 10 or more years. Only 66 (25.8%) participants had FBG below 126 mg/dl while 42 (16.4%) had a FBG of 250 mg/dl or more. Albuminuria was detected in 99 (38.8%) of the respondents (Table 1).

Of one hundred and nineteen (46.5%) hypertensive participants, 41 (34.5%) were undiagnosed. Sixty (23.4%) of the participants were obese. Of 197 participants whose lipid levels were determined, 125 (63.5%) were dyslipidemic. Low HDL-C was the commonest lipid abnormality, found in 99 (50.2%) of the participants. Smoking is the least frequent risk factor (Table 2).

**Table 2:** CVD risk factors among diabetic patients on follow up at JUSH, Oct – Dec, 2007.

CVD risk factors	Number	Percent
Hypertension	119	46.5
Obesity	60	23.4
Dyslipidemia (n = 197)	125	63.5
Current Smoking	14	5.5
Physical inactivity	141	55.1

**Figure 1:**
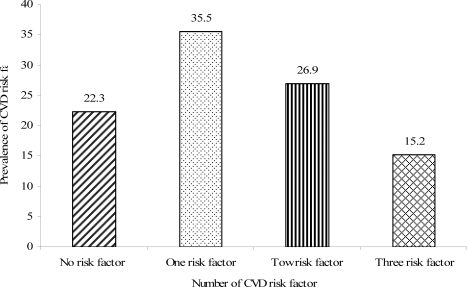
Frequency of multiple CVD risk factors (hypertension, obesity, dyslipidemia, smoking) in diabetic patients, JUSH, Oct – Dec 2007 (n = 197)

Among the 197 participants for whom all measurements were available, 153 (77.7%) had at least one of the four risk factors (hypertension, obesity, dyslipidemia, smoking). Seventy (35.5%) had only one risk factor while 30 (15.3%) had 3 or four factors (Fig. 1).

Age, gender, type of DM and obesity were found to be independently associated with hypertension. Hypertension was more than two times likely in those with age 45 years or older (OR=2.26, 95% CI; 1.06–4.82), three times in type 2 diabetics (OR=3.37, 95% CI; 1.16–8.18) and nearly four times in obese (OR=3.79, 95% CI; 1.72–8.36) patients than their respective counterparts. Females were 48% as likely to be hypertensive (OR=0.48, 95% CI; 0.23–0.99) as males. Address, educational status, duration of diabetes, type of treatment for diabetes, physical activity, smoking, alcohol use and chat chewing were not significantly associated with hypertension (Table 3).

**Table 3:** Multivariate analysis of the associations between selected characteristics and the CVD risk factors in diabetic patients, JUSH, Oct – Dec, 2007.

Independent variables		Hypertension OR(95% CI)	Obesity OR (95% CI)	Dyslipidemia OR (95% CI)
Age in years	< 45 ≥ 45	1 2.26(1.06–4.82)	1 1.04(0.44–2.42)	1 0.99(0.36–2.74)
Gender	Male Female	1 0.48(0.23–.99)	1 4.17(1.94–8.97)	1 4.25(1.69–10.68)
Address	Urban Rural	1 0.59(0.28–1.22)	1 0.37(0.16–0.87)	1 0.99(.39–2.53)
Education	≤ Primary ≥ Primary	1 0.84(0.38–1.86)	1 0.63(0.26–1.53)	1 1.16(0.45–3.00)
Type of DM	Type 1 Type 2	1 3.37(1.16–8.18)	1 6.08(1.52–24.23)	1 1.68(0.48–5.89)
Duration of diabetes	< 5 years ≥ 5 years	1 1.28(0.69–2.36)	1 1.02(0.49–2.10)	1 0.55(0.26–1.17)
Current treatment	Oral anti-DM Insulin	1 0.99(0.45–2.17)	1 1.06(0.47–2.41)	1 0.61(0.23–1.62)
FBG level	< 180mg/dl ≥ 180mg/dl	1 0.75(0.41–1.38)	1 0.56(0.27–1.16)	1 3.56(1.67–7.63)
Chat chewing	No Yes	1 0.92(.43–1.99)	1 1.49(0.57–3.89)	1 1.45(0.60–3.51)
Alcohol use	No Yes	1 0.77(0.37–1.60)	1 0.89(0.38–2.08)	1 0.39(0.17–0.89)
Physical activity	Yes No	1 1.4(0.72–2.73)	1 2.05(0.89–4.74)	1 1.45(0.64–3.26)
Current smoking	No Yes	1 0.32(0.07–1.45)	1 0.78(.08–7.43)	1 2.09(0.39–11.3)
Obese	No Yes	1 3.79(1.72–8.36)	*	1 0.85(.29–2.50)
Hypertension	No Yes	*	*	1 3.5(1.55–7.90)

* - not applicable

Type 2 diabetes and the female gender were the strongest independent predictors of obesity with OR (95% CI) 6.08(1.52–24.23) and 4.17(1.94–8.97) respectively. Rural subjects were 37% as likely to be obese as urban residents with (OR=0.37, 95% CI; 0.16–0.87). Other factors were not found to have significant association with obesity (Table 3).

Gender, FBG level, hypertension and alcohol use were independently associated with dyslipidemia. Dyslipidemia was more than four times common in females compared to males (OR = 4.25, 95% CI; 1.69–10.68). FBG level ≥ 180mg/dl was associated with nearly fourfold risk of dyslipidemia compared to lower levels. Hypertension also increased the risk of dyslipidemia by more than three fold (OR = 3.5, 95% CI; 1.55 – 7.90). Patients who drink alcoholic beverages were less likely to be dyslipidemic compared to those who didn't (OR = 0.39, 95% CI; 0.17–0.89). Other factors were not independently associated with dyslipidemia (Table 3).

## Discussion

Hypertension, obesity and dyslipidemia were common among diabetic patients in JUSH while cigarette smoking was uncommon. More than three fourth of the diabetic patients had at least one of the above cardiovascular risk factors. Age ≥ 45, type 2 DM, and obesity were predictors of hypertension. Type 2 DM and female gender predict obesity. Female gender, hypertension and FBG ≥ 180 mg/dl were predictors of dyslipidemia.

The prevalence of hypertension in this study is higher than the one reported (36.3%) among diabetic patients in JUSH in 2005 (13). This difference could be due to the inclusion of under 18 diabetics in the previous study. In our study, more than a third of the hypertensives were undiagnosed. Blood pressure was controlled in only 9.2% of all hypertensive cases. This is lower than findings in other studies carried out in USA (31%) and Spain 35% (9, 15, 18). This difference can be explained by differences in the study settings.

In this study; age ≥ 45 years, type 2 DM and obesity were positive predictors of hypertension consistent with findings from other studies (9, 10, 11, 18, 19). In the USA, 19.4% of type 1 and 74 % of type 2 diabetics had hypertension (9). The Diabetes in Germany (DIG) study showed the strong association of hypertension and obesity among type 2 diabetic individuals (p<0.001) (10).

Central obesity was more than twice as frequent as overall obesity (BMI>30 kg/m2) in this study. BMI of all of the obese individuals (by waist circumference) was > 25 kg/m2. Since overweight individuals with central obesity have similar CVD risks as those with BMI of 30–34.9 kg/m2 (20), considering them obese is appropriate. Jansen *et al* have shown that obesity related health risks are explained by waist circumference and not BMI (21). Many studies have shown that waist circumference predicts CVD risk similar to Waist to Hip Ratio (WHR) (20, 21, 22) and better than BMI and some point out that its simplicity makes it more useful than WHR (22).

In our study, 23.4% of the participants were obese which is lower than the NHANES III report where 51.6% of type 2 diabetics were obese (9). Other studies have also demonstrated that obesity is common in type 2 DM (10, 19, 23). Comparison is difficult because of the difference of the study subjects and definitions of obesity. However our study showed the association between type 2 DM and obesity consistent with other studies (9, 10, 23).

Female gender and type 2 DM were found to be positive predictors of obesity while rural residence was protective. The association between the female gender and obesity among diabetics is consistent with other studies (9, 10, 19, 23). The lower risk of obesity among rural residents can be explained epidemiologic transition that results from urbanization (24).

Nearly two-thirds of the participants were dyslipidemic. Low HDL-C and elevated triglyceride were more frequent while raised total cholesterol and LDL-C were less common. The high prevalence of dyslipidemia might be due to poor glycemic control among our study subjects. Poor glycemic control is associated with low HDL-C level and hypertriglyceridemia (6, 25). Compared to the study conducted at Tikur Anbesa Hospital (TAH) in 2003 where cholesterol level was elevated in almost half of the patients (26), our study showed lower frequency of elevated total cholesterol level. The difference may be due the fewer number of diabetic patients (100) included in the TAH study.

Female gender, hypertension and FBG ≥ 180 mg/dl are positive predictors for dyslipidemia. The association of dyslipidemia with female gender (6, 9) and poor glycemic control is consistent with other studies (25). We don't have any explanation for the association observed between hyperetension and dyslipidemia which requires further research. Alcohol users were less likely to be dyslipidemic. This might be because alcohol increases the level of HDL-C (16). However, further studies are needed to clarify this finding.

This study has several limitations. Since it is hospital based, the findings may not be applicable to the general population. Errors of measurement are possible but we have used training, pretest and supervision to ensure data quality. Hypertension and dyslipidemia might be overestimated by use of single measurement but we used standard operational definitions.

We recommend routine screening and management of hypertension, obesity, and dyslipidemia for diabetic patients in JUSH. Further studies aimed at assessing the impact of these CVD risk factors are needed. Similar studies in the community setting are needed.
